# LABRADOR—A Computational Workflow for Virus Detection in High-Throughput Sequencing Data

**DOI:** 10.3390/v13122541

**Published:** 2021-12-18

**Authors:** Izabela Fabiańska, Stefan Borutzki, Benjamin Richter, Hon Q. Tran, Andreas Neubert, Dietmar Mayer

**Affiliations:** IDT Biologika GmbH, Am Pharmapark, 06861 Dessau-Roßlau, Germany; stefan.borutzki@idt-biologika.de (S.B.); benjamin.richter@idt-biologika.de (B.R.); hon.tran@idt-biologika.de (H.Q.T.); andreas.neubert@idt-biologika.de (A.N.)

**Keywords:** adventitious virus testing, high-throughput sequencing, bioinformatics workflow, virus classification

## Abstract

High-throughput sequencing (HTS) allows detection of known and unknown viruses in samples of broad origin. This makes HTS a perfect technology to determine whether or not the biological products, such as vaccines are free from the adventitious agents, which could support or replace extensive testing using various in vitro and in vivo assays. Due to bioinformatics complexities, there is a need for standardized and reliable methods to manage HTS generated data in this field. Thus, we developed LABRADOR—an analysis pipeline for adventitious virus detection. The pipeline consists of several third-party programs and is divided into two major parts: (i) direct reads classification based on the comparison of characteristic profiles between reads and sequences deposited in the database supported with alignment of to the best matching reference sequence and (ii) de novo assembly of contigs and their classification on nucleotide and amino acid levels. To meet the requirements published in guidelines for biologicals’ safety we generated a custom nucleotide database with viral sequences. We tested our pipeline on publicly available HTS datasets and showed that LABRADOR can reliably detect viruses in mixtures of model viruses, vaccines and clinical samples.

## 1. Introduction

Production of biologicals, such as viral vaccines, is prone to adventitious, unintentionally introduced contaminants [1]. A potential contamination of vaccines poses a serious safety risk for human health and any contaminant incidence could highly affect a general public trust in the reliability of biologics. Testing for presence of adventitious viral agents (AVT) is particularly challenging as the viral contamination can be overlooked using in vivo or in vitro assays due to the incompatibility to the host cells or lack of visible infection [2,3]. PCR-based methods, although exhibiting high sensitivity of viral nucleic acids detection [4,5], can detect only a set of viruses for which the primers are designed and the reaction conditions are optimized [6]. Considering that nucleic acid sequence to design primers might be unavailable for viruses with poorly annotated genomes or for novel viruses, the potential contaminating virus can be missed with PCR-based methods. This obstacle can be overcome with high-throughput sequencing (HTS), which can detect any DNA or RNA molecule from biological sample regardless its intrinsic sequence. With HTS, it was possible to detect a porcine circovirus in rotavirus vaccine [7], for which a plausible contamination source was trypsin, necessary for growth and maintenance of Vero Cell Banks, a cell substrate used in production of this vaccine [8]. This example emphasizes a need for sensitive and specific detection of all viruses present in raw materials and pharmaceutical products at various stages of the manufacturing process [9].

The difficulties of HTS application for testing of biologics safety include the lack of standardization in sample and sequencing library preparation, necessary bioinformatics expertise and access to sufficient computational resources and storage space [10]. The selection of bioinformatic tools and their parameter optimization, adjusted to the sequencing platform used, have a major impact on viruses detection [11]. Raw sequencing data can suffer from low quality, so the read filtering algorithms have to deployed [12,13]. The computational pipeline’s core is the assignment of the taxonomic labels to the sequences in the dataset, which is performed using alignment-based or alignment-free methods, the latter being computationally less demanding and faster [14]. There are multiple well-tested alignment tools available, such as BLAST, BLAT, BWA or Bowtie2. Selection of the best one depends on the characteristics of the data (e.g., DNA/RNA, short/long reads) [15,16], but may be inconvenient in the case of viral genomes due to their high mutation rates, horizontal gene transfer and gene gains or losses [17]. The alignment-free classification can be carried out with programs like LMAT, Clark or Kraken [18,19,20] which are based on k-mers (nucleotide subsequences of length k) generation and comparison with the patterns generated from the sequence database. The major disadvantage shown of this type of classification is that it often results in several false positives, thus the extraction and comparison of discriminating patterns (k-mers) represents a field for algorithm’s improvement [21].

Another challenge of virus detection from HTS data is a curation of database with nucleotide and/or amino acid sequences. The database completeness and accuracy have a direct impact on the assignment of sequences based upon sequence identity to viral sequences in the database. The attempt of comprehensive viral database establishment has been undertaken in developing a Reference Viral Database (RVDB) based on semantic selection criteria to include all viral (except bacterial viruses), viral-like, and viral-related sequences, regardless of length and species, and with an overall reduced cellular content [22]. Another example is the Virosaurus database containing the viral sequences infecting eukaryotes, curated by clustering at 90% or 98% identity to remove redundant sequences [23]. Additionally, to nucleotide-based, databases with protein sequences can be used. Querying the sequences at the protein level can improve the detection of more distant relationship, like viruses divergent to currently deposited in the databases [24]. A protein version of RVDB was recently developed [25]. Continuous curation and extension of viral sequences in public repositories will support the HTS-based virus detection in the nearest future.

Publicly available bioinformatics workflows for handling viral HTS data can be divided according to the main research question into workflows for elucidating virus community (virome) composition or virus discovery. Examples of publicly available workflows oriented to virome composition comparison between samples include MetaVir2 [26], MetaShot [27] or ViromeScan [28]. Among the computational pipelines for virus discovery PathSeq [29], VirusHunter [30], VirusDetect [31] or Lazypipe [32] can be listed. Some pipelines such as VirusSeeker [33] can perform both virus composition study and discovery in two separate workflows, which makes it challenging to merge and compare the results of both approaches.

Generally, the workflows must include a taxonomic classification of reads or de novo assembly of longer sequences (contigs) and their subsequent taxonomic classification. The comparison of short reads against the database with reference sequences may fail to classify viruses if the matching identity is low. In this case assembling overlapping reads de novo into longer contiguous sequences could be helpful [34]. Most of de novo assemblers utilize one of two algorithms: overlap layout consensus (OLC) or de Bruijn graph. Assembler tests using viral metagenomics datasets have shown the limitation in time and RAM efficiency as well as being hampered by high coverage sequences in the case of OLC-based assemblers [35]. The most popular and well performing de Brujin assemblers used for virome datasets include MetaSPAdes, MEGAHIT and IDBA-UD [35,36,37]. If the entire genome analysis is the main interest of the study, a draft genome can be produced by joining the contigs together in a reference-guided alignment, i.e., alignment to a related viral reference sequence or alignment-free by scaffolding the contigs into the correct linear order [38].

From perspective of biologics’ safety, both ability to detect novel viruses and quantitative estimation of viral community members in the sample matters. However, compared to environmental studies, the relative-abundance representation of a sample is less valuable, as e.g., for attenuated vaccine samples the dominant species will be a vaccine virus, but it is foremost important to report all potential viral contaminants even of low initial concentration or fragmentation of nucleic acid. Thus, our main motivation for designing a new computational workflow tailored for biologics included a detection of segmented and whole genome viruses focusing especially on the viruses recommended by regulatory agencies in guidelines for biological safety testing. Moreover, we wanted to customize reporting of results generated by different bioinformatics tools for easy comparison between the samples. 

Here were present LABRADOR—a complete workflow for detection and precise classification of viruses in HTS datasets generated from biologics together with a custom nucleotide database. We named our workflow after the scent dog, Labrador, because dogs of this breed can be trained to discriminate between samples of virus infected and non-infected patients [39,40]. LABRADOR is written in Python and incorporates several open-source tools. It detects viruses using a pattern-based classification of sequencing reads coupled with mapping to corresponding nucleotide sequence, thus allowing the detection of low concentration or short fragments of viruses. In the complementary approach, de novo assembly and contig classification against nucleotide and amino acid databases is implemented to encounter for potential novel viruses. The viruses detected in both approaches are finally merged according to virus taxonomic identifier, facilitating the result comparisons. Moreover, to improve accuracy of sequence classification and customize it for biologics, a viral nucleotide database was generated for viral taxa recommended in guidelines for safety testing [41,42,43,44]. LABADOR could efficiently classify viruses from four published datasets i.e., in silico generated microbiome, spike virus experiments, vaccines and clinical samples, proving its comprehensiveness for virus detection. 

## 2. Materials and Methods

### 2.1. Software Environment

LABRADOR runs on a VMware based virtual machine (VM) with Red Hat Enterprise Linux 8.2 as the guest operating system. The VM is set up with 8 cores and 64 GB RAM. All bioinformatics tools were installed using Conda 4.9.0. A LABRADOR analysis of biologics sample of 10 M of 2 × 75 Illumina paired-end reads (PE) takes approx. 150 min.

### 2.2. LABRADOR Wrapper

The LABRADOR workflow is launched through a master script both written in Python 3.7.6. A graphical user interface (GUI) implemented with PySimpleGui 4.29 enables submission of input FASTQ files and other experimental settings. The GUI displays a progression bar, the corresponding computational process description and a message informing about the analysis end.

### 2.3. Preprocessing

Short and low-quality reads are removed with Trimmomatic 0.39 [45] using a sliding window of 25 nt, min quality of 20, and the read length threshold of 35 nt. High-quality reads are aligned to the host genomes with BOWTIE2 2.4.1 [46]. RefSeq genomes of green monkey, human and chicken are selected as standard host genomes (GCF_015252025, GCF_000001405.39 and GCF_000002315.6, respectively). Unmapped reads are extracted using SAMTools 1.10 [47] and converted to FASTQ format using BEDTools 2.29.2 [48]. 

### 2.4. Classfication of Viral Sequencing Reads and Mapping to Reference Genome 

After the host sequences removal, two approaches to classify viral sequences are undertaken. In the first approach non-host reads are classified with Kraken2 2.0.9 [49], a k-mer-based classifier with a default k-mer size against a custom viral nt database as a reference. The output taxonomic files (Kraken reports) are then reformatted into the MetaPhlAn-style text files using kreport2mpa function of Kraken Tools 0.1 (https://github.com/jenniferlu717/KrakenTools/, accessed on 25 September 2020) and Pandas 1.0.5 to retrieve a complete taxonomy path of classified viruses. For each classified viral LCA (lowest common ancestor: species or subspecies), the taxonomic identifiers (taxids) up to family level are extracted and used as input for the extract_kraken_reads function of Kraken Tools to extract reads assigned to these identifiers. For these taxids, the custom nt viral database is searched (Linux grep tool) for the matching reference sequences for mapping. To this end, the cluster with FASTA sequences collected in the custom viral database corresponding to the taxonomic identifier is screened for sequences of min 60% length of the longest sequence present in the cluster to avoid mapping to short sequences or incomplete genomes. Length of sequences is calculated with Biopython 1.78. For these FASTA sequences the representative compressed sketches are generated with MASH 2.2.2 sketch function [50]. The estimation of resemblance between the sketches generated for FASTA sequences and for reads extracted per taxid is performed with MASH screen command [51]. Finally, a FASTA sequence with the highest shared hash number with the reads is selected as a reference, for which sequence name and length are collected with SeqIO function of Biopython. If the reference sequence cannot be selected by this search, the central sequence of the cluster is considered the best reference. In the next step, host-depleted reads are aligned to the reference sequence(s) using BWA MEM 0.7.17 [52]. The number of mapped PE reads and the sequencing depth per genome position are retrieved with SAMTools 1.10 using the flagstat and depth commands, respectively. The sequencing depth per genome position is used to calculate the percentage of coverage of the reference sequence as: (number of positions with depth >0 × 100%)/length of reference sequence. Moreover, kurtosis and area-under-the curve (AUC) are calculated from coverage data with scipy 1.5.2 and sklearn 0.23.2 Python packages, which are used as additional parameters for estimating sequence coverage in metagenomics data as proposed by Aziz et al. (2015) [53]. The parameters describing coverage of reference genome are collected in table for each classified viral species and are further used to support the decision about true positive viral hits by a scientist. 

### 2.5. De Novo Contig Assembly and Classification

**Figure 1:**
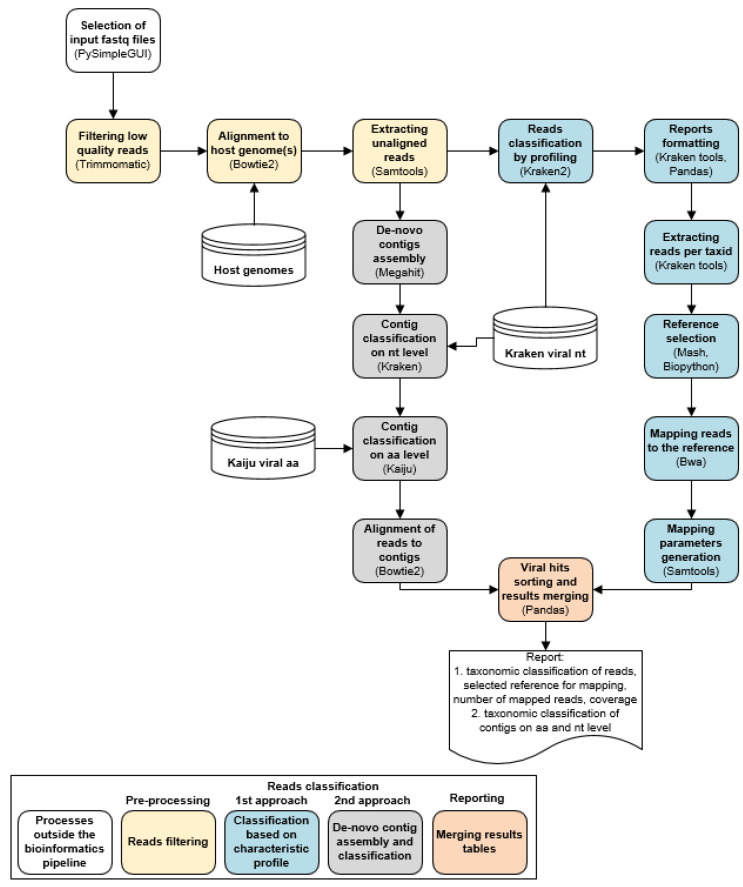
Workflow of LABRADOR workflow. Two approaches of viral sequences classification are highlighted in blue and grey.

In the second approach of the LABRADOR workflow, the reads, which passed host sequences filtering are assembled into longer sequences (contigs) with MEGAHIT 1.2.9 [54] Li et al., 2015) with minimal size of 500 nt (Figure 1, processes highlighted in grey). 

The resulting contigs are classified with Kraken2 against the custom nucleotide database and with Kaiju1.7.3 [55] using a translated search against amino acid (aa) database with default settings. The RVDB version of a database from 25 May 2020 is used (http://kaiju.binf.ku.dk/). Further, the reads after the host sequences filtering are aligned to contigs using Bowtie2 and the number of mapped reads is calculated with SAMTools. 

For each classified virus, the information from both approaches is merged in tables on the taxonomic identifier assigned by Kraken2 using Pandas. The information about viruses classified only in the second approach with possible novel viruses with amino acid sequences similar to viruses deposited in RVDB protein database is collected in the separate tables.

### 2.6. Creation of a Custom Viral Database 

Viral taxonomic groups recommended in the guidelines (24 families, 3 orders, 4 genera, Table S1) were collected and the NCBI database of viral genomes (https://www.ncbi.nlm.nih.gov/genomes/GenomesGroup.cgi, accessed on 13 March 2020) was searched to extract the genome accessions of viruses belonging to these groups. Subsequently, their complete genomes were downloaded from RefSeq [56] (12,145 sequences downloaded on 13 March 2020) and RVDB databases ver.18 [22] and treated as central points in collecting the FASTA sequences with 98% sequence identity from NCBI database in March 2020. In this way, 12,050 clusters were generated. From these clusters the viruses, which infected other than vertebrate hosts according to the NCBI collection of complete genomes and ViralZone [57], were removed. For FASTA sequences of final clusters, a custom nucleotide database was generated with Kraken2 using standard commands: “add-to-library” and “build” [49]. 

### 2.7. Evaluation of LABRADOR Workflow on Published Dataset

The pipeline was evaluated using previously published datasets from: (i) MetaShot project (in silico simulated microbiome data) [27], (ii) multicenter study performed to evaluate the HTS for virus detection [58], (iii) vaccine samples [7] and (iv) pneumonia patients [59] (Table S2). MetaShot dataset [27] contains 20.7 M 2 × 150 Illumina PE reads were generated, from which 19,582,500 are human (94.5%), 986,114 bacterial (4.8%) and 146,886 viral (0.7%) PE reads. According to the recent mapping of these reads against NCBI taxonomy and the Critical Assessment of Metagenome Interpretation (CAMI) taxonomic profile, this dataset contains 84 species and 46 genera of viruses [32], and this was used as a standard for taxa present in this dataset. The virus species and genus names were extracted from the taxonomic path of Kraken2 classification and were used for qualitative comparisons with MetaShot. Pearson’s correlation coefficient between reads mapped to reference using the LABRADOR pipeline and reads generated in MetaShot project was calculated in Python with corrcoef function for common viral species found in both datasets. 

From the multicenter study, we used the data generated by spiking four model viruses into HeLa cells or cell lysate done in the independent laboratories B and C [58]. The spiking viruses included viruses of different genome and particles characteristics, namely: Epstein-Barr virus (EBV, syn. Human herpesvirus 4), Feline leukemia virus (FeLV), Reovirus 1 (REO1, syn. Mammalian orthoreovirus 1) and Human Respiratory Syncytial Virus (RSV, syn. Human orthopneumovirus) added at three concentrations of 100, 3 or 0.1 genome copies per HeLA cell (high, medium and low concentration, respectively, Table S4). In experiments performed by Lab B, the viruses were spiked into two sample matrices: cell lysate and whole cells, and the sample preparation for sequencing was nonspecialized, capturing both RNA and DNA viruses. In contrast, in Lab C the viruses were spiked into whole cell matrix only, but the DNA and RNA material was extracted separately. For RNA samples, the rRNA depletion was performed to reduce cellular sequences. The samples were sequenced with Illumina HiSeq1500 in (Lab B) or HiSeq2500 (Lab C). 

To test LABRADOR on real samples, the data from additional published studies were used. Dataset of vaccine samples, originate from Victoria et al., 2010 study where 454 GS FLX technology was used to generated single-end reads for eight live-attenuated vaccines: Rotateq (Merck), Rotarix (GSK), Biopolio (Bahrat Biotech), Meruvax (Merck), Attenuvax (Merck), Varivax (Merck), YF-VAX (Sanofi) and MMR-II (Merck) [7]. The last dataset contained paired-end reads generated with MiSeq 3000 for nine samples collected from patients with severe pneumonia symptoms at the early stage of COVID-19 outbreak in China [59]. A detailed annotation and the source of sequencing data used are provided in Table S2. 

## 3. Results

### 3.1. Analyses Perfomed in LABRADOR Workflow and Custom Database Constuction

LABRADOR workflow can be divided into three main processes described in detail in the Materials and Methods section. Entire workflow is written in a single Python script starting with user interface for FASTQ data submission, through automated analysis and classification of sequences, until collecting and merging the results in tables (Figure 1). In the preprocessing step, low-quality and host-origin reads are filtered. Currently, LABRADOR removes reads of green monkey, human and chicken origin, covering the most commonly used continuous cell lines for the production of vaccines and viral vectors [60,61,62]. After read processing, the non-host reads are used to detect viruses in two complementary approaches. In the first one, reads are directly classified with Kraken2, and the reads classified to the lowest common ancestor (species or subspecies) are summed up with the higher ancestors up to family level, extracted and used for searching the matching reference genome from custom nucleotide database. The family level was selected considering that the taxonomic profiling accuracy of Kraken program may decrease below family level [63]. After aligning reads to the reference, the details of reference sequence are collected and the mapping parameters are calculated: number of mapped reads, length of reference genome covered, coverage percentage, density and uniformity (Figure 1, Table S3). 

In the complementary approach of LABRADOR software, non-host reads are assembled *de novo* into longer contigs with MEGAHIT (Figure 1). The contigs are then classified using Kraken2 against our custom nucleotide database to have a basis for results comparison with the first LABRADOR approach. Additionally, contigs are also classified with a complete protein RVDB, to detect distant viral taxa, considering that substitution rates in amino acid are much lower than in nucleic acid sequences and in order to detect viruses that might not be included in our custom database. The results from both approaches are then joined according to the taxonomic identifiers originating from Kraken2 classification into single table and in the case of *de novo* only detection, additional tables are generated. The result table thus contains: Kraken2 virus classification, selected reference sequence, mapping parameters and contig classification on nt and aa levels. The conclusion about the viruses present in the sample must take into account the name of the matching reference and its coverage and the classification on the nt and levels. In addition, the sequencing sample preparation has to be considered e.g., knowing that the virus enrichment with biotinylated probes was performed, it can be expected that in the first LABRADOR’s approach the low genome coverage would suggest a virus presence and that some viruses could be missed in the second LABRADOR’s approach where 500 nt long contigs are generated. 

**Figure 2:**
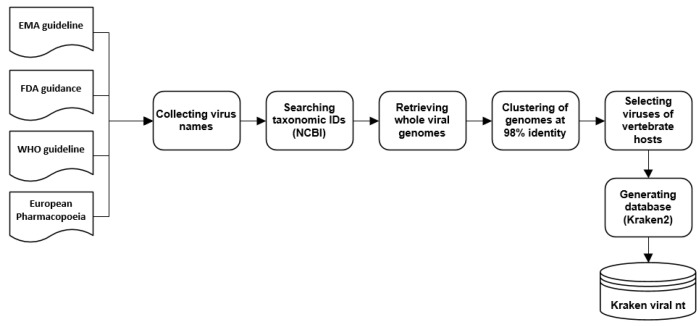
Flowchart of viral database generation. The database includes nucleotide sequences of viruses listed in the guidelines for biologic safety and known to infect vertebrates (Table S1).

To classify viral sequences using the k-mer- based approach of Kraken2 [49] we generated a customized nucleotide database (Figure 2) containing sequences of viral taxa recommended in guidelines for safety testing of biologicals published by the European Medicines Agency (EMA), Council of Europe, World Health Organization (WHO), or the United States Food and Drug Administration (FDA) [41,42,43,44]. From these documents we extracted all viral taxa mentioned and searched the NCBI database for the sequence identifiers of viruses belonging to these taxonomic groups. After downloading the whole genomes of all taxa, they were used for clustering against NCBI database at 98% identity, to collect the published nucleic-acid information of related viral isolates matching the given taxa (species). To facilitate the analysis of biologics, only viruses infecting vertebrates were considered relevant to generate a final database (Table S1). This resulted in 3008 clusters of guideline viruses, with 672,402 nucleotide sequences in total.

### 3.2. LABRADOR’s Performance on Simulated Metagenomics Dataset

Simulated in silico datasets allow to compare the pipeline classification performance excluding the bias introduced by sample preparation in the laboratory. Thus, we conducted a LABRADOR analysis on a simulated human microbiome from MetaShot project [27,32] and filtered out the viral hits, for which no contig could be classified in the *de novo* assembly approach (Table S3).

Overall, a list of detected viruses in LABRADOR pointed that the taxonomic classification of viruses using both approaches implemented in the pipeline highly resembled each other (Table S3). Moreover, the contig classification with our viral custom database resulted in similar taxonomic names as the ones obtained with Kaiju program and aa RVDB. This confirms that our custom viral database covers a wide range of viruses that could be found in a standard human metagenome. The selection of a reference for the reads mapping using MASH implemented in the LABRADOR pipeline performed well overall, resulting in selection of more than one reference if the reads equally matched more sequences from the database as e.g., for Dengue Virus (four serotypes 1, 2, 3, 4 selected) or for West Nile virus (lineage 1 and 2). However, for segmented viruses, some discrepancy in selection of the best reference sequence could be observed e.g., in the case of Rotavirus A reads, for three out of ten segments the retrieved reference sequence was originating from another subspecies (Human rotavirus A, Simian rotavirus A) as the other seven segments (Rotavirus A segments 1–4, 6, 9, 10). A high coverage of viral references was observed (average 97.0% ± 7.17), without dependency between number of mapped reads and the percentage of reference coverage (Pearson’s correlation coefficient 0.078). For instance, for Influenza A, for which there were only 90 reads, 98.9% of reference sequence was covered (Table S3). This shows that LABRADOR can also classify viruses with low sequencing depth. 

**Figure 3:**
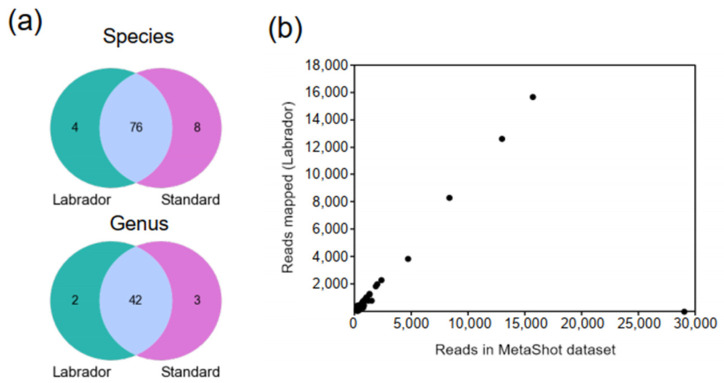
Accuracy of virus classification with the LABRADOR pipeline assessed on the MetaShot dataset containing the simulated microbiome reads published by Fosso et al. (2017) [27]. (**a**) Number of viral species and genera classified by LABRADOR and found in taxonomic standard profile generated for MetaShot dataset [32]. (**b**) Number of reads that were classified to viral species found by LABRADOR or generated in silico for the MetaShot dataset. Reads mapped to reference sequence in 1-st approach of LABRADOR workflow (classification based on reads characteristic profile) were considered (Table S3).

A qualitative analysis of results was performed by assessing presence or absence of viral taxa at two taxonomic levels (species and genus) taking the CAMI taxonomic standard as a reference [32]. LABRADOR classified 80 viral species and 44 genera, corresponding to 90% and 93% of taxa present in Metashot dataset according to CAMI classification (Figure 3a). LABRADOR showed high precision (reflecting the proportion of actually correct positive identifications among all positive identifications) of 95% and 95.5%, at the species and genus level, respectively (Table 1). Pipeline’s recall value, which is synonymous to sensitivity, was 90.5% at the species, and 93.3% at the genus level (Table 1). This shows that in comparison with other computational pipelines, LABRADOR detects higher (compared to older pipelines) or similar (compared with the recent Lazypipe pipeline) number of positive viral hits as well as finds relatively low number false negative hits. Viral species missed in LABRADOR analysis were the ones which were not present in the database i.e., PhiX bacterial virus (absent both in custom database and RVDB) and human endogenous viruses, whose sequences were filtered out in the host removal step. Other discrepancies between LABRADOR and CAMI taxonomic standard profile for Fosso et al. (2017) [27] dataset included the viruses, which were classified to different but corresponding viral species, e.g., Adeno-associated dependoparvovirus B (standard) and Bovine adeno-associated virus (LABRADOR), Human bocavirus (standard) and Primate bocaparvovirus 1 (LABRADOR). Overall, this shows high reliability of virus taxonomic classification provided in LABRADOR.

The quantitative evaluation of LABRADOR was performed by checking if it outputs similar number of reads classified as a given virus as actually present in the dataset. To this end, for viral species present in the Metashot dataset and classified by LABRADOR, the number of reads generated in the original MetaShot dataset was plotted against number of reads mapped to reference genomes in LABRADOR (Figure 3b). The number of reads in LABRADOR highly mirrored the number of reads in the dataset, however one outlier point with 29,018 reads in the Metashot dataset and six reads in LABRADOR could be seen. This is due to the human endogenous virus, for which six reads were mapped to the reference genome. According to the original publication [27], the dataset contained 32,541 reads of human endogenous viruses. Running the LABRADOR pipeline without the host removal step has yielded 32,690 reads mapped to the retroviruses, which highly resembles the number of reads in the dataset. Thus, the low number of human endogenous virus reads was due to the host reads removal performed prior reads classification. We correlated the number of reads mapped to the reference sequences in LABRADOR analysis with the number of reads in the MetaShot dataset (for common viral species found in both datasets, Figure 3b). Pearson’s correlation coefficient was 0.562 considering the outlier value of human endogenous virus and 0.997 when the outlier was removed. Therefore, it can be stated that the number of reads mapped to the reference genome selected in the LABRADOR pipeline effectively estimates the real read number in the dataset, except for viruses that are parts of the host genome. 

### 3.3. Detection of AVT Model Viruses from Spiking Study

To test LABRADOR on the experimental data with known viruses, we used the datasets generated in two laboratories (B and C) taking part in the multicenter study to evaluate HTS for AVT, where three concentrations of four model viruses were spiked into different cellular matrices simulating the materials used in production of biologics (whole cells and cell lysate) [58]. LABRADOR analysis of samples from labs B and C resulted in detection of all spiked viruses in Lab B samples with high spike concentration into cell lysate and whole cells, Lab B sample with medium spike into whole cells and in all RNA samples of Lab C (Table S4). REO1 was not detected at medium and low spike into both cell matrixes, whereas FeLV at the low spike concentration into cell lysate or whole cells prepared in lab B. As it could be expected, the RNA viruses (REO1, FeLV, RSV) were not detected in DNA samples generated in Lab C. Additionally, Human papillomavirus type 18 (HPV18), which is integrated in the HeLa genome was detected in all samples from both laboratories with the coverage spanning 58–67% of the reference genome. The detection of spike viruses and HPV18 with LABRADOR resembled the results obtained with the bioinformatics analyses performed in the respective laboratories in the original study [58]. 

The de novo approach performed in LABRADOR resulted in no contig for viruses with the number of sequencing reads below 50. Still, in samples with lower concentration of spike viruses, the viruses could be detected with direct reads taxonomic assignment with Kraken2, indicating the complementary effect of both methods implemented in the LABRADOR pipeline. The different methods of classification used in the LABRADOR pipeline could result in different viral hits at the taxonomic level below species (strain or serotypes). For instance, for REO1 virus with 10 segments in the genome, the references found for seven out of 10 segments originated from serotype 1, corresponding to the classification of all de novo assembled contigs on the amino acid level pointing to the serotype 1 of this virus (Table S4). For two segments, the best matching references originated from subtype 3 of REO virus, while for segment S1 of REO1, a S1 segment of Mammalian orthoreovirus 4 Ndelle has shown the best match. The further read mapping, resulted in up to 100% (in high spike sample) or 57.5% (in low spike sample) of the sequence coverage for 1 and 3 subtypes, while no read was aligned to segment 1 of serotype 4. This example highlights that especially for segmented viruses, LABRADOR’s results have to be interpreted considering all classification methods and mapping statistics, since LABRADOR correctly points to the virus species but the conclusion about the serotype can be made based on the best mapping results and predominant classification of contigs. 

### 3.4. Detection of Viruses in Datasets from Real-Life Experiments 

The LABRADOR pipeline was finally used to analyze two real-life experiments with FASTQ files originating from (i) vaccine samples of different producers (Victoria et al., 2010), (ii) patients with severe pneumonia at the early stage of the COVID-19 outbreak [59]. In the case of first dataset, the single-end sequencing technology was implemented, thus we generated the corresponding reverse FASTQ file prior analysis. Generally, the expected attenuated viruses could be detected in the vaccine samples (Table S5). Non-vaccine porcine circoviruses (PCV) were present in Rotarix vaccine. The number of mapped reads to PCV1 genome was 6032 and translated in a complete coverage of PCV1 genome (100%), whereas for PCV2 2956 reads were mapped and 56.4% genome was covered. De novo contig assembly resulted in 66 contigs classified as PCV1 and two as PCV2. Additionally, aside from pipeline inspection of contigs and mapping their sequences against PCV genomes resulted in coverage of 100% for PCV1 and 52.3% genome coverage of PCV2, further confirming the high genome integrity of PCV1 and lower integrity of PCV2 viruses present in Rotarix vaccine. 

Analysis of the dataset containing reads from pneumonia patients samples resulted in classifying with Kraken2 in eight out of nine samples a severe acute respiratory syndrome coronavirus 2, for which a genome of the Wuhan seafood market pneumonia virus isolate Wuhan-Hu-1 was selected for mapping reference using a MASH screen (Table S6). The genome coverage of SARS-CoV-2 virus was high and ranged from 74.3–100%. Furthermore, also with high genome coverage (88.8–96.7%), Influenza virus A could be detected in two samples. Additionally, in two samples Human endogenous retrovirus K was be found with coverage of 54.3% and 60.6%, suggesting that some human reads remained after the host sequences removal. Overall, analysis of real-life experimental data confirms that the LABRADOR pipeline can be used to detect viruses in datasets from broaden origin spanning biologicals and human clinical samples.

## 4. Discussion

The computational data analysis as well as the coherence and accuracy of the reference database highly affect the outcome of HTS studies [10,22]. We developed LABRADOR, a computational workflow for virus detection in HTS datasets together with the customized database containing nucleotide sequences of viruses recommended for the evaluation of biological products. At the beginning of our pipeline, a simple quality filtering reads is performed with a well-established tool, Trimmomatic [45], because we examine the quality of our internal sequencing runs with another tool prior to using LABRADOR. However, if the quality of input FASTQ files has to be proven in greater detail, especially when the pipeline is used to analyze files of different origin, other QC tools, such as AfterQC [12] or FastqCleaner [64], can be implemented. Taxonomic classification in LABRADOR is then performed by querying the reads and assembled contigs, which allows the identification of viruses for which only a fraction of a genome is present. 

Compared to other available pipelines, such as ViromeScan [28] and Metashot [27], which align high-quality reads to the database, LABRADOR takes advantage of reads classification using a k-mer-based approach. To this end, our tool of choice was Kraken2, which was shown to outperform the alignment-based methods or ranked in the top of alignment-free methods in high classification speed, low memory usage and high accuracy of species assignment [18,21,49]. This accuracy is reflected in the high proportion of true positive hits at species level (Figure 1) confirming the ability of LABRADOR to detect the viruses at high taxonomic resolution. The virus that LABRADOR cannot detect in tested datasets is a human endogenous virus in the MetaShot dataset, which is due to the human reads removal performed in preprocessing step. Generally, the differentiation between host sequences and endogenous retroviruses would require another as the HTS-based assays for example a product-enhanced reverse transcriptase (PERT) assay [65]. 

Implemented in LABRADOR search of the reference sequence based on the comparison of representative compressed sketches for reads and for FASTA sequences deposited in the custom database (MASH), performed overall well considering the high coverage of the reference after alignment to the reference (Tables S3–S6). Nevertheless, in some cases, e.g., for viruses with segmented genomes, the selected references for some genome segments originated from another viral serotype. Still, the conclusion about the virus presence based on LABRADOR’s results must also consider the classification performed on the contig level. This function is often implemented in the pipelines for virus discovery e.g., Lazypipe [32] or VirusDetect [31] to identify viral genomes with lower similarity to known viruses deposited in the database. Utilized in LABRADOR, a MEGAHIT assembler belongs to the top performing de Bruijn graph assemblers and could also find low coverage genomes of a metagenome [54,66,67]. In analyzed datasets, a dozen of viral reads (<100) was enough to build contigs of min 500 nt length and reliably classify them (Tables S3–S6), confirming suitability of MEGAHIT for low coverage genomes. 

The precision of LABRADOR with our custom database was higher compared to Centrifuge or MetaPhlan2 (32% and 10.5% higher, respectively) and similar to Kraken2 with standard database or Lazypipe computational pipelines (95.5% LABRADOR, 94.1% Kraken2, 90% or 97.2% for Lazypipe depending on classification method used) as shown recently for the simulated microbiome [32]. The recall value at the species level obtained with LABRADOR was higher than for MataPhlan2, Kraken2 (45.3%, 71.5% higher, respectively) and Lazypipe (8.4% or 4.8% depending on the classification method selected in Lazypipe, Table 1) [32]. The high accuracy and sensitivity of LABRADOR resulting in correct identification of viruses actually present in the data are especially important for detection of adventitious agents in biologicals as it can help to prevent releasing a potentially harmful product for patients. This was further corroborated by results obtained for vaccine datasets as similar to Victoria et al. (2010) [7]; LABRADOR analysis pointed to the presence of porcine circoviruses in the Rotarix vaccine (Table S5). This implies that LABRADOR workflow can be used for HTS-based AVT. 

Khan et al. (2017) [10] compared independent workflows, including sample preparation and bioinformatics analyses, for the capacity to detect four model viruses of different genome and particle characteristics. In these datasets, LABRADOR detects similar number of reads (considering the reads mapped to the reference genome) for the spike viruses as shown by Labs B and C, despite differences in the bioinformatics methods used (Khan et al., 2017). Correspondingly, the genome coverage reported by LABRADOR resembles the ones presented by Lab B and C in the original study (Table S4). With the sample preparation used by Lab C including the depletion of rRNA, it can be concluded that LABRADOR can detect 0.1 viral genome copies per cell, which then corresponds to 62–664 mapped reads of RNA viruses (REO1, FeLV, RSV) or 366 reads of DNA viruses (HPV). However, a detailed limit of detection (number of viral genome copies per ml or host cell) for the LABRADOR pipeline will have to be estimated based on the sequencing data generated for samples prepared with a well-established laboratory method. To this end, the genetic material of viruses with distinct genome characteristics and particle sizes should be included. 

Detection of SARS-CoV-2 virus in eight out of nine samples and Influenza A in two samples from Wuhan is in line with the results obtained with Lazypipe HTS pipeline. Lazypipe HTS, which also implements de novo contig assembly with MEGAHIT (similar to LABRADOR’s de novo approach) and further contig classification with Centrifuge against NCBI nt database or SANSparallel against UniProtKB database (Table S6, [32]). Compared to results of Plyusnin et al. (2020) and Zhou et al. (2020) [32,59], LABRADOR does not detect the viruses of insect, fungal (Saccharomyces) or plant hosts, which stays in accordance with our custom viral database content including only the viruses hosted by vertebrates. This confirms the utility of the LABRADOR pipeline to characterize real-life human clinical samples. 

Considering the advantages of HTS e.g., its sensitivity and decreasing costs of sequencing, it will probably replace current methods for adventitious virus detection [10]. The parameters collected for the viruses found in LABRADOR analysis (Tables S3–S6) can highly support the decision about the positive viral hits based on the number of reads classified, coverage of reference genome and contigs classified, knowing the sample preparation process and individual characteristics of viruses. However, the follow-up experimental strategy will have to be developed to further confirm the presence of adventitious virus in the sample [68]. There is also a need to develop representative samples to enable the comparing of different laboratory assays for adventitious virus detection prior sequencing [6]. Submitting the HTS datasets of such samples to public repositories would increase the scarce availability of FASTQ files from biologicals and will be helpful to evaluate performance of bioinformatics tools or completeness of viral databases developed for AVT assays.

## Figures and Tables

**Table 1:** Precision and recall values for the Metashot dataset. The precision and recall values for Centrifuge, Kraken2, and Lazypipe were published by Plyusnin et al., 2020 [32].

Taxomic Level	Tool	Precision [%]	Recall [%]
Species	LABRADOR	95	90.5
	Lazypipe-nt	97.2	82.1
	Lazypipe	90.0	85.7
	Centifuge	63.0	95.2
	MetaPhlan2	84.4	45.2
	Kraken2	94.1	19.0
Genus	LABRADOR	95.5	93.3
	Lazypipe-nt	95.3	91.1
	Lazypipe	95.3	91.1
	Centifuge	84.9	100
	MetaPhlan2	88.9	71.1
	Kraken2	95.5	46.7

## Supplementary Materials

The following are available online at https://www.mdpi.com/article/10.3390/v13122541/s1, Table S1: Viral taxa found in guidelines for biologics safety and accession numbers of viruses belonging to these taxa, Table S2: Datasets used to test LABRADOR’s performance, Table S3: Viruses detected with the LABRADOR pipeline in simulated in silico microbiome dataset, Table S4: Viruses detected with the LABRADOR pipeline in datasets generated in two laboratories by spiking model viruses in HeLa cells, Table S5: Summary table of viruses detected with the LABRADOR pipeline in vaccine samples, Table S6: Summary table of viruses detected using the LABRADOR pipeline in clinical samples of pneumonia patients.
